# A comparative study on the Efficacy of Retroperitoneoscopic Pyeloplasty and Open Surgery for Ureteropelvic Junction Obstruction in Children

**DOI:** 10.12669/pjms.37.7.4205

**Published:** 2021

**Authors:** Jianghua Jia, Qingsong Meng, Ming Zhang, Jinchun Qi, Dongbin Wang

**Affiliations:** 1 Jianghua Jia, Department of Urology, The Second Hospital of Hebei Medical University, NO. 215 Heping Xi road, Shijiazhuang, Hebei, 050000, China; 2 Qingsong Meng, Department of Urology, The Second Hospital of Hebei Medical University, NO. 215 Heping Xi road, Shijiazhuang, Hebei, 050000, China; 3 Ming Zhang, Department of Urology, The Second Hospital of Hebei Medical University, NO. 215 Heping Xi road, Shijiazhuang, Hebei, 050000, China; 4 Jinchun Qi, Department of Urology, The Second Hospital of Hebei Medical University, NO. 215 Heping Xi road, Shijiazhuang, Hebei, 050000, China; 5 Dongbin Wang, Department of Urology, The Second Hospital of Hebei Medical University, NO. 215 Heping Xi road, Shijiazhuang, Hebei, 050000, China

**Keywords:** Retroperitoneoscopic pyeloplasty, Open pyeloplasty, Ureteropelvic junction obstruction, Children

## Abstract

**Objectives:**

To compare the therapeutic effect of retroperitoneoscopic dismembered pyeloplasty and open ureteropelvic junction plasty on the ureteropelvic junction obstruction (UPJO) in children.

**Methods:**

After the retrospective analysis of clinical data, 78 children with ureteropelvic junction stenosis treated from January, 2012 to June, 2018 were divided into two groups: OP (open pyeloplasty) group (38 cases) and LP (laparoscopic dismembered pyeloplasty) group (40 cases) according to the surgical methods. The operation time, intraoperative bleeding volume, postoperative length of stay (LOS), postoperative complication rate, postoperative hydronephrosis improvement and other indicators were compared between the two groups.

**Results:**

All patients underwent surgery successfully, without conversion to open surgery in LP group. The incidence of postoperative urine leakage and the recovery of hydronephrosis between LP group and OP group 12 months after operation showed no statistically significant difference (P>0.05). The intraoperative bleeding volume, the incidence of postoperative retroperitoneal hematoma, and the postoperative LOS in LP group were lower than those in OP group, while the operation time was longer than that in the OP group, with statistically significant difference (P<0.05).

**Conclusion:**

Retroperitoneoscopic dismembered pyeloplasty had similar effect with open dismembered pyeloplasty, but faster recovery and fewer complications, so it has become the preferred treatment method for UPJO in children.

## INTRODUCTION

Ureteropelvic junction obstruction (UPJO) is the main cause of hydronephrosis in children, and early diagnosis and timely treatment are of great significance to the protection of renal function of children. If children’s hydronephrosis is not treated or treated incorrectly, renal function will be further impaired. There are a variety of surgical methods for the treatment of UPJO, of which dismembered pyeloplasty is considered to be the gold standard for the treatment of UPJO with the success rate of over 90% and good long-term efficacy^1^,^2^ after long-term follow-up. After more than 20 years of development, laparoscopic pyeloplasty has been widely used for its advantages of less pain, less trauma, faster recovery, better cosmetic effect and higher success rate than open surgery. The technique improvement has made retroperitoneoscopic pyeloplasty feasible and effective, even for the infants with UPJ obstruction, and our preliminary experience report is as follows.

## METHODS

The procedures followed in this study comply with the ethics standards established and approved by the ethics committee, and all subjects and their parents have given informed consent. The UPJO children hospitalized from January, 2012 to June, 2018 were selected as the study subjects, and divided into OP group (open pyeloplasty) and LP group (laparoscopic dismembered pyeloplasty) according to surgical methods. Among the 38 children in OP group, there were 25 males and 13 females, aged from 12.3 to 73.2 months, with an average age of (35.6±23.8) months, and there were 26 cases of moderate hydronephrosis and 12 cases of severe hydronephrosis. Among the 40 children in LP group, there were 25 males and 15 females, aged 10.5-80.9 months, with an average age of 37.4±22.5 months, and there were 25 cases of moderate hydronephrosis and 15 cases of severe hydronephrosis. The two groups of children were compared in terms of age, sex, severity of hydronephrosis and other basic conditions, and all the P values were >0.05, which was comparable (see Table-I). Both groups received preoperative intravenous urography (IVU), urinary ultrasound and CT. IVU showed delayed development time of renal pelvis and calyx on the affected side, dilatation of renal sinus, and abrupt termination of contrast agent at the ureteropelvic junction, the ultrasonography showed hydronephrosis signs but no ureteral dilatation, and the preoperative blood and renal function examination remained within the normal range.

**Table I:** Comparison of the general information of Children in OP group and LP Group.

*Index*	*OP group (n=38)*	*LP group (n=40)*	*p*
Age (month)	35.6±23.8	37.4±22.5	0.956
***Sex***			
Male	25	25	0.873
Female	13	15	
***Affected side***			
Left	26	24	0.438
Right	12	16	
***Hydronephrosis degree***			
Moderate	26	25	0.583
Severe	12	15	

**Table II:** Comparison of the intraoperative and postoperative follow-up data of the children in LP group and OP group.

*Item*	*OP group (n=38)*	*LP group (n=40)*	*p*
Time of operation/min	114.4±13.1	122.7±13.4	0.007
Intraoperative bleeding volume/ml	29.4±9.7	21.9±7.4	<0.001
Postoperative LOS	8.9±1.3	7.1±1.2	<0.001
Incidence rate of leakage of urine/% (cases)	5.6(2/36)	11.1(4/36)	0.433
Incidence rate ofretroperitoneal hematoma/% (cases)	15.2(5/33)	0	0.018
***Postoperative follow-up recovery after 12 months***			
No hydronephrosis	33	34	0.815 0.745 0.958
Mild hydronephrosis	3	4	
Moderate hydronephrosis	2	2	
Severe hydronephrosis	0	0	

The retrospective analysis study was approved by the Institutional Ethics Committee of The Second Hospital of Hebei Medical University treated from January, 2012 to June, 2018, and written informed consent was obtained from all participants.

### Retroperitoneal laparoscopic surgery

**Fig.1:**
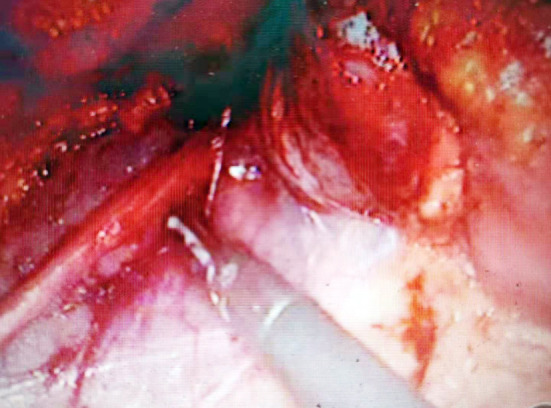
Dissociate UPJ.

**Fig.2:**
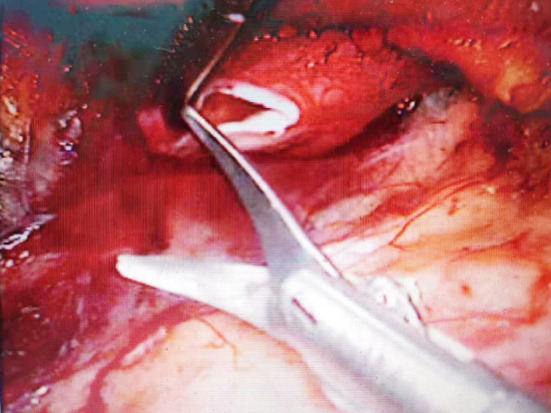
Cut UPJ.

**Fig.3:**
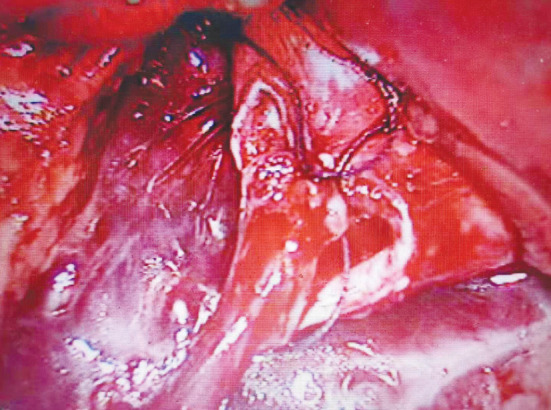
Anastomose UPJ.

**Fig.4:**
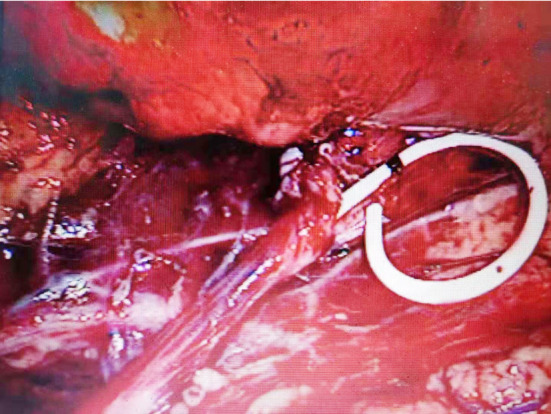
Place Double J tube.

**Fig.5:**
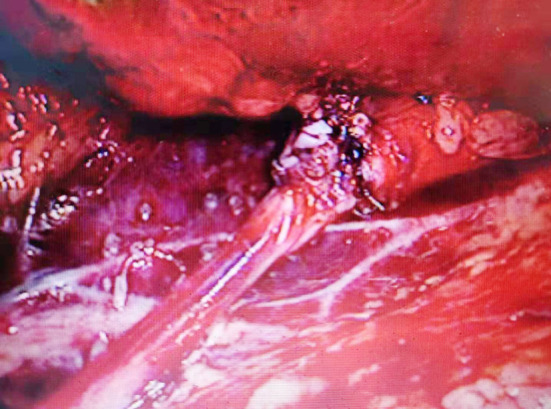
Finish anastomosis J.

After general anesthesia, a catheter was indwelled and clamped, and then the patient was aligned waist and raised waist bridge in the unaffected side-lying position, and connected to the corresponding laparoscopic device after routine disinfection and surgical drape spreading. An incision of about 2.0cm was made below the costal margin of posterior axillary line, which was opened to the lumbar dorsal fascia layer by layer, and then broken through into the retroperitoneal space. A visual balloon was inserted, inflated with about 300ml-360ml air, and then removed after five minutes. Suture fixation was performed after 10 mm Trocar cannula was embedded, and then the laparoscope was inserted and the pneumoperitoneum was connected, with the pressure maintained at 8-10 mmHG, and the incision was made at the anterior axillary line below costal margin, midaxillary line, and two transverse fingers above ilium; 5 mm and 10 mm Trocars were inserted under laparoscope, with the core withdrawn, and the laparoscope moved to Trocar cannula above axillary midline and ilium. The elastic separating plier and ultrasonic knife were inserted into the remaining two Trocar cannulas respectively. The perirenal fascia was opened, ranging from subphrenic space to iliac fossa. The perirenal fat was opened to separate the space between anterior psoas major and posterior kidney, so that the free upper ureteral segment and dilated renal pelvis was under direct vision. The renal pelvis was cut in an arc, so that the renal pelvis was bell-opened, and the ureter of stenotic segment was cut off about 0.5cm from the distal end of stenotic segment. The lateral proximal ureteral wall was cut longitudinally by about 1.0cm. The lowest point of flared opening of renal pelvis and the lowest point of the cut of ureter was sutured with 4-0 absorbable needle suture for one stitch, while the highest point of flared opening of renal pelvis and the highest point of the broken end of ureter was sutured with 4-0 absorbable needle suture for one stitch. Continuous suture of posterior wall was performed with 4-0 absorbable suture, one locking stitch every two stitches, the distal end of double J tube was inserted into urinary bladder at anastomotic stoma guided by the thread, which was drawn later, ensuring the urine could flow out from the side hole when the abdomen was squeezed, while the proximal end was inserted into renal pelvis, and 4-0 absorbable suture with needle was used to suture the anterior closed anastomotic stoma, and then the closed urethral catheter was opened. After no bleeding, errhysis and urine leakage could be observed at the anastomotic stoma, and peristaltic ureteral waves could pass through the anastomotic stoma, the retroperitoneal drainage tube was placed, and then the incision was sutured layer by layer (Fig.1-5). The drainage tube was removed 2~5 days after the operation, the urinary tube was removed 7~10 days after the operation, and the ureteral double J tube was removed by ureteroscopy under general anesthesia 6~8 weeks later.

### Open Surgery

The urinary catheter was placed in the urinary bladder after successful general anesthesia. The patient was placed in the healthy side-lying position with the waist slightly elevated, and then an oblique incision was made at the waist in the affected side after routine disinfection and surgical drape spreading, and the skin was incised layer by layer to subcutaneous tissue, obliquus externus abdominis, obliquus internus abdominis and musculus trasversus abdominis. The peritoneum was opened and the perinephric fascia was cut open to make the pelviureteric junction and upper ureteral segment fully exposed, and the indication lines was drawn inside and outside the renal pelvis and ureter respectively, and then the renal pelvis was cut open above the stenosis segment, which was cut arc-shaped to make the renal pelvis outlet flared, then the stenosis segment was removed, and the normal ureter was incised longitudinally about 1.0 cm under the stenosis segment; afterwards, the anastomosis was performed between pelvis outlet and proximal ureter with 4-0 absorbable surgical suture: anastomosed the anterior wall firstly, placed the ureteral stent (with the distal end passing through anastomotic stoma and the proximal end led in vitro via pelvis incision), led in the pyelostomy catheter through skin incision, and then place it in the renal pelvis through another pelvis incision, and then anastomosed the posterior wall. One retroperitoneal drainage tube was indwelling and the incision was sutured layer by layer. The drainage tube and urinary catheter were removed two to five days after the operation, the ureteral stent was removed 10~14d after the operation, and the nephrostomy tube was removed after methylene blue was injected one to two days after the operation to confirm the ureteral patency.

### Observation indexes

The operation time, intraoperative bleeding volume, postoperative LOS, and postoperative complication rate (incidence of urine leakage and retroperitoneal hematoma) of the children in the LP group and OP group were compared and observed. Intraoperative blood loss was calculated by the total amount of fluid aspirated minus the amount of intraoperative rinse fluid. The follow-up visits were made to the two groups of children at the 3^rd^, 6^th^ and 12^th^ months after the operation respectively, and the recovery was observed through B-ultrasound examination of urinary system, with the number of severe, moderate, mild and no hydronephrosis cases in the two groups at the 12^th^ month as the statistical indicators. The degree of hydronephrosis was determined by the urinary tract dilation(UTD) classification system^3^: (1) mild: 1.0~2.0cm separation of collecting system, and normal renal parenchyma and kidney shape; (2) moderate: 2.1~3.5cm separation of collecting system, slightly thinner renal parenchyma, and enlarged kidney shape; (3) severe: more than 3.6cm separation of collecting system, significantly thinner renal parenchyma, and enlarged and deformed kidney shape.

### Statistical Method

SPSS16.0 statistical software was used for statistical analysis, with the measurement data expressed as X ±S, two independent samples were used for t-test, and the counting data were tested by chi-square test, P <0.05 indicating statistically significant difference.

## RESULTS

All patients received surgery successfully, without conversion to open surgery in the LP group. The incidence of postoperative urine leakage and the hydronephrosis recovery between LP group and OP group 12 months after surgery showed no statistically significant difference (P>0.05). The intraoperative bleeding volume, the incidence of postoperative retroperitoneal hematoma, and the postoperative LOS in LP group were lower than those in OP group, while the operation time was longer than that in the OP group, with statistically significant difference (P<0.05). The duration of indwelling drainage tube and LOS were prolonged for the children with postoperative leakage of urine and retroperitoneal hematoma.

## DISCUSSION

It has been reported in most studies that the success rate of open Anderson–Hynes pyeloplasty is greater than 90%, so it is the preferred method for surgical treatment of pediatric UPJ obstruction^4^. Minimally invasive surgery has become a growing trend over the past decade. The small retroperitoneal space in pediatric patients is not conducive to the operation of laparoscopic instrument, so the application of retroperitoneal laparoscopic technology in pediatric urological surgery is limited.

In 1999, Tan et al.^5^ reported that 18 children underwent laparoscopic pyeloplasty via peritoneal approach and two underwent secondary surgery for the first time. Yeung et al.^6^ also reported that 13 infants underwent laparoscopic pyeloplasty via retroperitoneal approach in 2001, one of which was converted to open surgery. Zhou huixia et al.^7^ Summarized and reported 36 cases of children undergoing laparoscopic pyeloplasty via retroperitoneal approach, and believed that it is prone to dissociate after pneumoperitoneum formation and the anatomical hierarchy is clear for the children with little retroperitoneal fat and loose tissue; therefore, retroperitoneal laparoscopic pyeloplasty is a safe, effective and minimally invasive method for the treatment of pediatric UPJ stenosis.

The laparoscopic pyeloplasty can be performed via abdominal approach and retroperitoneal approach, each of which has its advantages and disadvantages.^8^-^10^ The operation space via abdominal approach is larger, and the surgical field is clear, but it is easy to cause intra-abdominal viscera injury, and postoperative complications, such as abdominal distension, intestinal obstruction, and intestinal adhesion, etc.; moreover, because the renal pedicle vessels make the ureteropelvic junction difficult to be exposed, thereby largely increasing the operation difficulty, while it is well exposed via retroperitoneal approach, which is convenient for operation, with small abdominal injury, and no significant complication. Retroperitoneal laparoscopic pyeloplasty is difficult, and skilled surgeons are required to reconstruct the renal pelvis and ureter under the endoscope; in addition, due to limited operating space, it is difficult to complete suture and knotting in vivo, and a certain learning curve is required. In this study, all the patients in the LP group were successfully operated without being converted to open surgery. The intraoperative bleeding volume, the incidence of postoperative retroperitoneal hematoma, and the postoperative LOS in LP group were lower than those in OP group, while the operation time was longer than that in the OP group. Rasool S et al also reported that intraoperative blood loss was significantly lower in the LP group than in the OP group.^11^ Another study also found that there are shorter operative times in the laparoscopic-assisted pyeloplasty and shorter overall hospitalization.^12^ There was no significant difference in the incidence of urine leakage and recovery of hydronephrosis between the two groups 12 months after operation. Our surgical experience is summarized as follows:

(1) The lumbar and dorsal fascia of children is immature, and the peritoneum is relatively thin, so the surgical operation should be gentle, and when the expansion balloon is used to prepare retroperitoneal space, the peritoneum should not be overinflated and torn. Generally, it should be inflated about 300-360ml, and the pneumoperitoneum pressure is set as 8-10mmHg.
(2) It is generally not dissociated in the kidney of ventral side, but only dissociated in the lower middle part of the dorsal side of kidney, and it will seek and follow the non-vascular plane for dissociation, so it is necessary to avoid injury and bleeding, especially in children, who have small blood volume, and it is essential to actively stop bleeding, so as to ensure a clear operating field;
(3) When placing the cannula, it is necessary to first place and fix the cannula at the incision of posterior axillary line, connect the pneumoperitoneum, place the laparoscope, and place another two cannulas under the laparoscope to avoid injury to retroperitoneum.
(4) The ureteral lumen in children is small and fine, so the posterior wall can be sutured with intermittent locking stitch during anastomosis. After the ureteral DJ tube is placed, the anterior wall can be sutured with intermittent 2~3 stitches, and the suture should not be too close, so as to reduce the incidence of postoperative anastomotic stenosis.
(5) Laparoscopic double “J” intra-tube drainage is performed with double drainage of luminal drainage and peri-tube drainage, which can effectively reduce the incidence of infection at the anastomotic stoma, reduce the incidence of recent incision infection and urine leakage, and effectively promote the recovery of patients.^13^

During laparoscopic surgery, it should be converted to laparotomy in time if the following conditions appear^14^,^15^: (1) severe adhesion between renal pelvis and surrounding tissues, unclear anatomical structure, and difficulty in laparoscopic separation and resection; (2) intraoperative calculus, and difficulty in thorough removal under the laparoscope; (3) intraoperative bleeding, and ineffective control under the laparoscope; (4) intraoperative injury of duodenum or colon, and difficult to make accurate repair under the laparoscope; (5) during the operation, the length of lesion to be removed is found to be longer, the tension of anastomotic site is high, it is difficult to perform accurate anastomosis, and there are few skilled surgeons for laparoscopic surgery.

Urethrovesical anastomotic leakage is the most common complication after pyeloplasty, which is usually due to inadequate laparoscopic anastomosis, postoperative regression of anastomotic edema, urinary extravasation, or stent blockage and displacement. Good technique of laparoscopic anastomosis, unblocked internal stent drainage, and indwelling catheter to keep bladder drainage at low pressure to prevent reverse flow can reduce the incidence of urine leakage. Generally, it can be cured by maintaining the peritoneal drainage tube smooth, and delaying the removal of drainage tube. If the postoperative urine leakage continues, the possibility of ureteral obstruction and stent displacement should be considered. If necessary, stent replacement or nephrostomy should be performed, and nutrition should be strengthened to promote wound healing, so that the condition can be improved 1-2 weeks later generally.^16^,^17^

Secondary obstruction after UPJO surgery is one of the major complications after pyeloplasty. Studies have shown that reoperation after primary pyeloplasty is required for about 11% of patients, indicating that the actual achievement rate of pyeloplasty is lower than that reported in the literature. The author believes that the key to the success of the operation is to determine the lowest point of renal pelvis and anastomose it with the ureter.^18^ Zhou Huixia^19^ et al. determined the direction of renal axis and the lowest point suture technique by using the direction of the upper, middle and lower calyx zaxis, so as to reduce the risk of postoperative re-obstruction, and improve the achievement ratio of operation; meanwhile, the non-clamp anastomotic technique can reduce the injury of anastomotic tissue and blood vessels, and improve the achievement ratio of operation.

## CONCLUSION

Retroperitoneal laparoscopic dismembered pyeloplasty share the similar effects with open dismembered pyeloplasty, but the faster recovery and fewer complications have made it the preferred treatment for UPJO in children.

### Limitations of this study

The number of subjects included in this study was limited, so the conclusions drawn may not be very convincing. In addition, we only analyzed and discussed the cases included in our hospital, which may not be representative enough. We look forward to a multi-center study in the future to reach more comprehensive conclusions.

### Authors’ Contributions:

**JJ** & **DW:** Designed and performed the experiments, wrote the manuscript, and are responsible and accountable for the accuracy or integrity of the work.

**QM**, **MZ** & **JQ:** Conducted the experiments and revised the manuscript. All authors have read and approved the final manuscript.
